# The intrinsically disordered protein glue of the myelin major dense line: Linking AlphaFold2 predictions to experimental data

**DOI:** 10.1016/j.bbrep.2023.101474

**Published:** 2023-04-26

**Authors:** Oda C. Krokengen, Arne Raasakka, Petri Kursula

**Affiliations:** a Department of Biomedicine, University of Bergen, Norway; b Faculty of Biochemistry and Molecular Medicine & Biocenter Oulu, Oulu, Finland

**Keywords:** Myelin, Intrinsically disordered protein, Membrane binding, AlphaFold2, Small-angle X-ray scattering, Circular dichroism spectroscopy, Conformation

## Abstract

Numerous human proteins are classified as intrinsically disordered proteins (IDPs). Due to their physicochemical properties, high-resolution structural information about IDPs is generally lacking. On the other hand, IDPs are known to adopt local ordered structures upon interactions with *e.g.* other proteins or lipid membrane surfaces. While recent developments in protein structure prediction have been revolutionary, their impact on IDP research at high resolution remains limited. We took a specific example of two myelin-specific IDPs, the myelin basic protein (MBP) and the cytoplasmic domain of myelin protein zero (P0ct). Both of these IDPs are crucial for normal nervous system development and function, and while they are disordered in solution, upon membrane binding, they partially fold into helices, being embedded into the lipid membrane. We carried out AlphaFold2 predictions of both proteins and analysed the models in light of experimental data related to protein structure and molecular interactions. We observe that the predicted models have helical segments that closely correspond to the membrane-binding sites on both proteins. We furthermore analyse the fits of the models to synchrotron-based X-ray scattering and circular dichroism data from the same IDPs. The models are likely to represent the membrane-bound state of both MBP and P0ct, rather than the conformation in solution. Artificial intelligence-based models of IDPs appear to provide information on the ligand-bound state of these proteins, instead of the conformers dominating free in solution. We further discuss the implications of the predictions for mammalian nervous system myelination and their relevance to understanding disease aspects of these IDPs.

## Highlights

• AlphaFold2 predicts MBP and P0 cytoplasmic tail structures as partially folded.
• The models were compared to experimental data on protein structure.
• The predicted folded structures likely represent the lipid membrane-bound state.
• AlphaFold2 may be valuable in predicting interacting conformations of flexible proteins.

## 1 Introduction

The artificial intelligence/machine learning-based algorithms of protein structure prediction, most notably AlphaFold2 [1] and RoseTTAFold [2], have recently revolutionised structural biology. AlphaFold2 is trained on crystal structures, which suggests it will predict conformations that one might find in a protein crystal, and for many folded proteins, the predictions are essentially identical to the crystal structure – sometimes even allowing error detection in the experimental structure [3]. With the development of AlphaFold2, the structural coverage of all human protein residues has significantly increased [4]. It is, however, obvious that for intrinsically disordered proteins (IDPs) and flexible multidomain proteins with intrinsically disordered regions (IDRs), AlphaFold2 cannot predict a single accurate 3D structure – which in such cases does not even exist.

IDPs or IDRs do not fold into stable 3D structures, but rather exist as an ensemble of conformations. Their conformational properties depend on their amino acid composition, and upon molecular interactions, secondary structures can form. IDR segments can also be described using the foldamer theory [5], as the distribution of hydrophobic and polar residues in their sequence determines their ability to perform order-disorder transitions and local folding. IDPs are, hence, physicochemically different from denatured globular proteins [6]. Due to their specific properties as polymeric chains, several biological functions have been attributed to IDPs and IDRs. These include, but are not limited to, acting as molecular rulers, forming membraneless organelles, protecting from collapse under plant dehydration [7,8], increasing the avidity of clamp binders [9], and binding to lipid membranes [10]. Conformational plasticity and the ability for context-dependent folding are central for the functions of IDPs.

Considering the above, AlphaFold2 predictions seem relevant for IDPs [11]. Firstly, AlphaFold2 predicts reliably regions that will not fold under any normal circumstances [12]. Secondly, it can predict segments that might fold upon binding to target molecules, *i.e.* the predicted local structure is that of the protein in complex with other proteins or lipid membranes. This context-dependent folding has been predicted by other bioinformatics tools before [13,14], allowing to detect functional regions in IDPs that interact with other molecules.

Since AlphaFold2 and RoseTTAFold became available, their relevance for IDPs has been actively discussed [11,[15], [16], [17]]. IDRs often undergo a disorder-to-order transition upon binding to an interaction partner [17], and IDPs can form secondary and tertiary structures upon binding, involving either the whole protein or only a short segment [18,19]. Even though IDPs remain a major challenge for structural biology, their importance raises interest in implementing AlphaFold2 as part of the workflow when analysing IDPs. AlphaFold2 produces a per-residue confidence score (pLDDT) between 0 and 100, signifying the confidence level of the prediction, being classified as very high (pLDDT >90), confident (90 > pLDDT >70), low (70 > pLDDT >50) and very low (pLDDT <50). These scores can be used to identify ordered/disordered regions in IDPs and IDRs [15,12,20]. pLDDT scores below 50 predict disorder and should not be interpreted as a structure; a score below 70 should be treated with caution [21,22]. Low pLDDT scores signify highly flexible residues, which still may contribute to protein function, but may also reflect lack of representation of such segments in databases used for machine learning [11,23].

Myelin is a multilayered proteolipid membrane in the central and peripheral nervous system (CNS and PNS, respectively), which is wrapped around selected axons by myelinating glia. The fast nerve conduction velocity enabled by the myelin sheath is mandatory for the normal functioning of the vertebrate nervous system. The compacted myelin membrane carries a unique set of proteins, which are either integral or peripheral membrane proteins that bind lipid bilayers together into multilayers. Myelinating cells express several specific IDPs, which are crucial for the correct formation and stability of the myelin membrane multilayer [24], such as myelin basic protein (MBP) and periaxin. Additionally, myelin protein zero (P0), the major structural protein of PNS myelin, contains a short cytoplasmic IDR (P0ct). The folding of disordered myelin proteins has been studied using both full-length proteins and peptide segments [[25], [26], [27], [28], [29], [30], [31]], allowing detection of membrane interaction sites and membrane-induced folding into helices.

MBP is one of the best-characterized proteins of the myelin sheath, playing a role in many interactions, oligodendrocyte proliferation, myelinogenesis and membrane stacking [32]. MBP changes conformation depending on its interactions and the chemical environment, and its disordered nature was described already nearly 50 years ago, well before IDPs had become a central topic in protein biochemistry [33]. Mendz et al. suggested in 1990 that certain interaction sites within MBP form helices when mixed with detergent micelles [34]. Especially this model has been considered for the central helical segment between residues 82 and 93 in mouse 18.5-kDa MBP (85–96 in human MBP), and an α-helical model would facilitate interactions with lipid head groups [35]. Electron paramagnetic resonance spectroscopy and molecular dynamics simulations revealed an amphipathic α-helical structure for this segment [36], penetrating up to 12 Å into a myelin-like membrane. Three MBP segments, T33-D46, V83-T92 and Y142-L154, have α-helical propensity; the formation of these helices is regulated by local hydrophobic interactions between the nonpolar surface of the helix and the lipid bilayer [37]. The interactions between MBP and lipid monolayers are also electrostatic, and the protein binds strongly with increased fraction of negatively charged headgroups [30].

P0 is the major protein in the PNS myelin, being primarily expressed in Schwann cells. The Ig-like extracellular domain has been structurally characterized in atomic detail using X-ray crystallography [38,39]. Within the myelin sheath, P0 is assumed to form homodimers *via* a glycine zipper in the transmembrane domain [40]. P0 molecules are believed to oligomerize between apposing membranes [41] *via* both the extracellular and intracellular domains. P0ct is not only important for membrane stacking at the PNS major dense line, but it could be involved in P0 trafficking, which is further regulated by post-translational modifications (PTMs) in P0ct [42]. P0ct is comprised of 69 residues, being disordered in solution and having a high positive charge. However, Charcot-Marie-Tooth (CMT) disease-causing mutations have been identified within this IDR [[43], [44], [45], [46], [47], [48]], highlighting its importance for proper myelination. Like MBP, P0ct folds into helical structures upon interactions with lipid membranes [49,50]. The folding was earlier suggested to be mostly β-sheets, but later studies strongly support α-helical conformation [50,51]. We showed that full-length P0 organises into zipper-like assemblies when reconstituted into bilayers, and P0ct in a lipidic environment produced Bragg peaks in X-ray diffraction [50], indicating spontaneous assembly of ordered, repetitive structures.

The high-resolution 3D structure determination of MBP and P0ct has proven to be difficult, if not impossible. Here, we extracted information through AlphaFold2 models of MBP and P0ct. For both proteins, we analyse earlier experimental data in light of the AlphaFold2 models and show that such models are valuable even in the case of highly flexible, disordered proteins, helping to understand the function and interactions of these proteins at the molecular level. Combination of small-angle X-ray scattering (SAXS) and synchrotron radiation circular dichroism spectroscopy (SRCD) data analysis indicates that the models from AlphaFold2 are more representative of the membrane-bound states than the free solution form of MBP and P0ct.

## 2 Methods

### 2.1 Generation of molecular models for MBP and P0ct

AlphaFold2 [1] was run on the Google ColabFold server [52], giving as input the amino acid sequence of mouse 18.5-kDa MBP isoform and human P0ct. The resulting 5 models were all relaxed with the Amber implementation in AlphaFold2. The models were used as such for further analyses.

### 2.2 SAXS data analysis

Synchrotron SAXS data for mouse MBP and human P0ct from our earlier publications [10,50] were directly used to assess the fits of the AlphaFold2 models to the experimental data. The data had been collected in batch mode using synchrotron radiation on the EMBL/DESY beamline P12 [53], at 1–4 mg/ml in 20 mM HEPES (pH 7.5), 300 mM NaCl, 1% (w/v) glycerol [10,50]. For data analysis and model fitting, the programs CRYSOL [54], OLIGOMER [55], and EOM [56] were used. R_g_ values were additionally estimated using the Guinier plot in PRIMUS [55] and with the Debye formalism, as described [57,58]. D_max_ was manually estimated using GNOM [59], such that the distance distribution had a reasonable shape and the fit to the raw SAXS data was optimal.

### 2.3 SRCD data analysis

The AlphaFold2 models were used as input for the PDB2CD software, which calculates theoretical CD spectra based on protein 3D coordinates [60]. The calculated CD spectra were compared with experimentally collected SRCD data previously published [10,50]. These data included MBP and P0ct both in solution and bound to lipid membranes, and they were collected from protein samples at 0.2–0.5 mg/ml in water. Both the proteins alone and mixed with 1:1 DMPC:DMPG liposomes at a protein/lipid molar ratio of 1:200 were analysed. In addition, P0ct was studied in the presence of 0.5% SDS detergent. Data had been collected [10,50] on the synchrotron beamlines UV-CD12 (KARA, KIT, Karlsruhe, Germany) [61] and AU-CD (ASTRID2, ISA, Aarhus, Denmark).

### 2.4 Bioinformatics and structure analysis

Sequence-based secondary structure predictions for both proteins have been published before [49,62]. The highest-scoring AlphaFold2 models of MBP and P0ct were docked as rigid bodies onto planar lipid bilayers with properties of a mammalian plasma membrane, using the PPM 3.0 server [63,64]. Default parameters for the PPM server were used in the docking; this represents a crude rigid-body docking with no atomistic information, and the membrane is considered a fluid anisotropic solvent. The docking is based on calculating the transfer energy from water to the membrane environment; details of the algorithm can be found in the original publication [63]. Visualization and surface electrostatics calculations were carried out in PyMOL [65] with the APBS [66] plugin.

## 3 Results and discussion

Inspired by work from others on understanding IDPs and their conditionally folded segments [15,16,21], we set out to analyse earlier experimental SAXS and SRCD data [10,50] as well as literature in light of AlphaFold2 models of MBP and P0ct. We expected to get an improved picture about the membrane-induced folding of these two myelin-specific IDPs, when they interact with membrane surfaces. AlphaFold2 can predict, which parts of the IDP do not fold upon interactions with other molecules or surfaces; these segments could promote “fuzzy” complexes [67] of MBP and P0ct on the membrane surface.

**Fig. 1:**
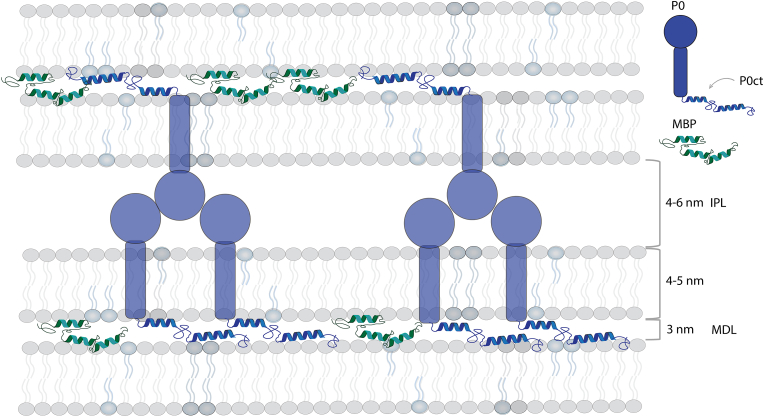
**Schematic view of PNS myelin, with MBP and P0 holding the stacked membranes together.** Note how P0 is adhesive on both sides of the membrane, and P0ct resides in the same compartment, the major dense line, as MBP. IPL, intraperiod line; MDL, major dense line.

For both MBP and P0ct, data from various biophysical experiments have been published [42,[49], [50], [51],[68], [69], [70], [71]], showing partial folding upon membrane binding, while both proteins remain unfolded in aqueous solution. The regions binding to membranes have been mapped to specific segments, mainly those prone to fold into helices according to secondary structure predictions. Several studies have shed light on more details of membrane binding by focusing on peptides corresponding to the membrane-binding sites. The relation of the membrane-binding sites of myelin proteins with possible autoantigenic epitopes in disease [25,62], such as multiple sclerosis, together with molecular mimicry of certain viruses like EBV [72], suggests that detailed fundamental studies on myelin protein-membrane interactions can give new insights into both myelin biology and pathology. How useful might an AI-based prediction of protein 3D structure be in this scenario? Fig. 1 shows a schematic overview of a PNS myelin multilayer held together by both P0 and MBP. We focus here on the molecular interactions at the major dense line, *i.e.* the tight apposition of two cytoplasmic membrane leaflets mediated by MBP and P0ct.

### 3.1 Myelin basic protein – the molecular glue in myelin

**Fig. 2:**
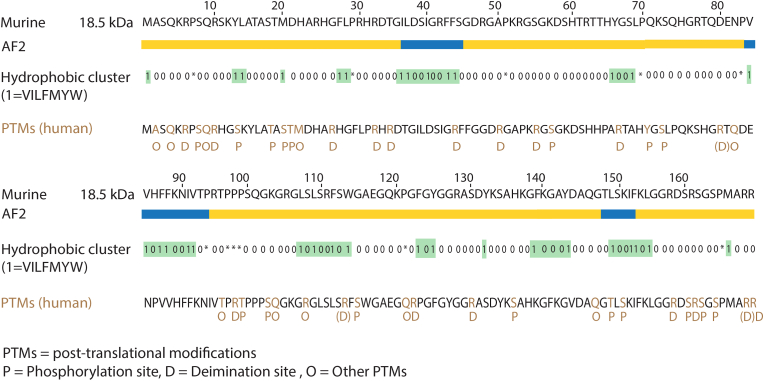
**The mouse 18.5-kDa isoform of MBP.** Below the sequence, the following are indicated: secondary structure of the AlphaFold2 model (blue – helix, yellow – disordered), the results of the hydrophobic cluster analysis (green – hydrophobic cluster), known PTMs [74]. (For interpretation of the references to colour in this figure legend, the reader is referred to the Web version of this article.)

The oligodendrocyte lineage (Golli) gene gives rise to a variety of MBP isoforms ranging from 14.0 kDa to 21.5 kDa, with the 18.5 kDa isoform being predominant in human mature myelin [37]. All isoforms of MBP have a positive charge depending on PTMs, referred to as C1 to C8, where C1 is the most basic isomer (net charge of +19 at physiological pH), with the least PTMs [73]. The most common PTMs are phosphorylation and citrullination (deimination), the C8 isoform being the least basic isomer, having decreased ligand interactions compared to the C1 isoform [74]. The basicity of MBP is required for its interaction with negatively charged phospholipids: in the less basic C8 isoform, the C-terminal region was reported to dissociate from the membrane, while the N-terminal site was more dynamic than in C1 [70]. The Phe86/Phe87 motif was important for the formation of the helix and its attachment to lipids [70,74], and mutation of these residues abolished phase separation *in vitro* [75]. The most abundant and experimentally by far the best-studied isoform of murine MBP, the 169-residue 18.5-kDa isoform (Fig. 2), was used for the analyses here.

**Fig. 3:**
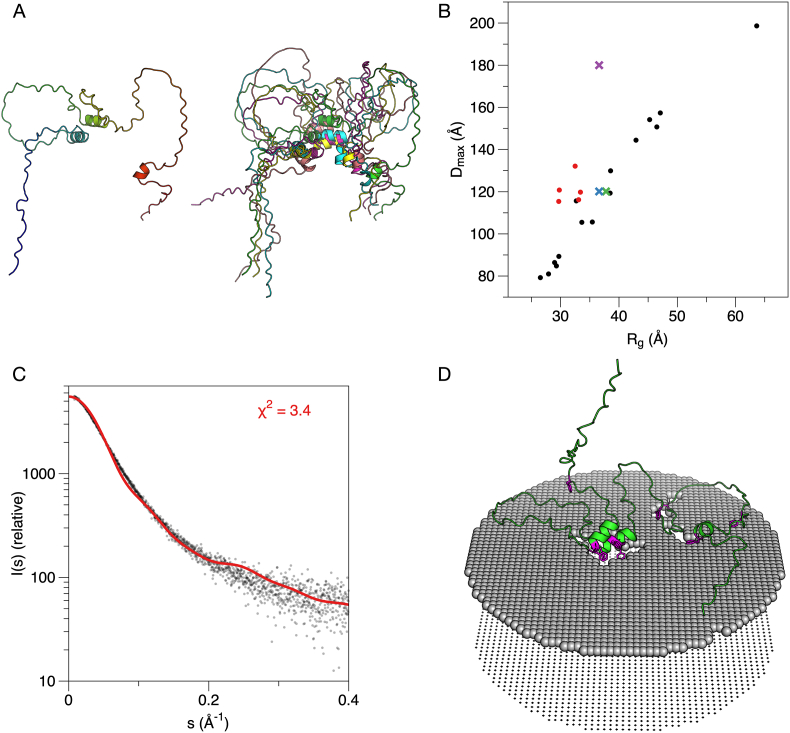
**AlphaFold2 models of MBP.** A. The top ranked (left) model and superposition of the 5 obtained models (right). Note how the overall dimensions and shape are similar, and that the three short helices cluster in the middle in all cases. B. Comparison of the AlphaFold2 models (red dots) and the EOM ensemble based on solution SAXS data (black dots). The average R_g_/D_max_ from EOM is shown with a green cross, the Guinier R_g_ with D_max_ from EOM with a blue cross, and the Guinier R_g_ with manually determined D_max_ from GNOM with a magenta cross. While the models cluster close to the average experimental values from EOM, they systematically have a lower R_g_, which is a sign of the presence of folded structure. C. Fit of the top ranked model alone to experimental SAXS data [10]. The sub-optimal fit is likely related to the secondary structures in the model and their clustering. D. Docking of the top ranked model onto a lipid bilayer surface suggests membrane interactions by the helices and supports the hypothesis that the predicted model reflects the membrane-bound state. Phe residues are highlighted in magenta. (For interpretation of the references to colour in this figure legend, the reader is referred to the Web version of this article.)

The five models of MBP predicted by AlphaFold2 are shown in Fig. 3A. All 5 models have similar folds and dimensions, with three predicted helices. The superposition of the obtained models creates a structural ensemble akin to those obtained *e.g.* from EOM based on SAXS data [76], but in this case, the models are based on sequence alone.

A more detailed analysis of the predicted structures is, therefore, warranted. A comparison of their R_g_ and D_max_ to those obtained from EOM is shown in Fig. 3B. The AlphaFold2 models apparently have a smaller R_g_ for the same D_max_, when compared to the flexible random-chain models produced by EOM – this reflects the presence of folded secondary structure elements in the models; also note how the predicted helices tend to cluster together in the models (Fig. 3A). Three helices are predicted in all models (at residue ranges 36–45, 83–92, and 148–153), and these correspond to the membrane- and calmodulin-binding sites identified earlier [[28], [29], [30],^74^] that become α-helical upon binding. Furthermore, same segments have been found to have strong α-helical propensity in NMR experiments [77,78]. This observation indicates that AlphaFold2 may have predicted, at least partially, the membrane-bound conformation of MBP, rather than the form free in solution.

The highest-ranked AlphaFold2 model of MBP provided a questionable fit alone to the raw experimental SAXS data (Fig. 3C). The fit was not improved by fitting all five models simultaneously using OLIGOMER (Table 1). This does indicate that the predicted structures do not appear to represent the conformation in solution. Indeed, we might expect AlphaFold2 to rather predict the membrane-bound model than the one free in solution. With this in mind, we docked the AlphaFold2 structure onto a membrane surface (Fig. 3D). Whether the three individual MBP helices cluster together on the membrane, as seen in the AlphaFold2 models, is not known; the model confidence scores are discussed further below. Notably, the two main helices that come together in the models both harbour a double Phe motif, which is crucial for MBP function in membrane stacking [75]. MBP compacts drastically upon being embedded between two membranes [10], which are only 3 nm apart, and such clustering of helical segments could be a mechanism of structural compaction. Given that the internal helices of MBP would cluster together as predicted by AlphaFold2, this could represent the intermolecular mechanism that ultimately results in the liquid-liquid phase separation of MBP, possibly even driving solvent exclusion from the membrane stack [75]. In addition, the conformation could coarsely represent the formation of a gel-like protein phase on the surface of a lipid bilayer, which we earlier observed using cryo-EM and neutron reflectometry [10]. This protein phase can subsequently attract a second bilayer, whereby MBP compacts between two cytoplasmic leaflets [10,79].

**Table 1:** **Fits of different models to experimental synchrotron SAXS data.** The values given in the table are χ^2^ for the fit between model and experimental data.

Protein	MBP	P0ct
Highest-ranked AlphaFold2 model alone	3.4	1.3
OLIGOMER solution, fitting all 5 AlphaFold2 models	4.3	1.3
Full EOM ensemble	1.0	1.0
Chain-like *ab initio* model (GASBOR)	1.1 [10]	1.1 [50]

### 3.2 The cytoplasmic domain of P0 – similar but different to MBP

**Fig. 4:**
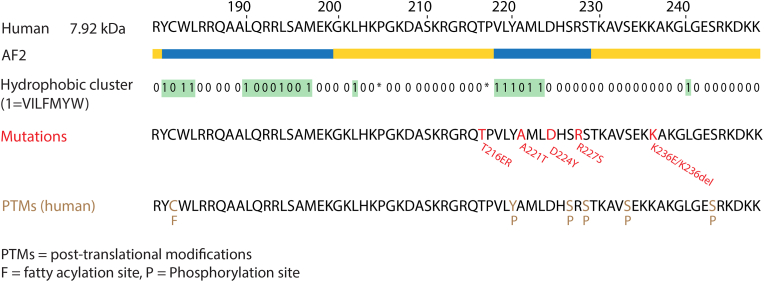
**The cytoplasmic domain of myelin protein P0.** P0ct spans between residues 180–248 of human P0. Below the sequence, secondary structures from AlphaFold2 as well as hydrophobic cluster analysis are shown like in Fig. 2. In addition, positions for known CMT mutations as well as PTMs are indicated.

P0ct has a strong (+15) positive charge, carrying 21 basic and 6 acidic residues [80] (Fig. 4). P0ct free in aqueous solution is unfolded, as determined by SRCD spectroscopy, but it gains helical secondary structure upon lipid interactions [49,50]. Of specific interest is the fact that CMT disease-causing mutations are found in P0ct [[43], [44], [45], [46], [47], [48]], which suggests an important function for this IDR in normal myelination.

**Fig. 5:**
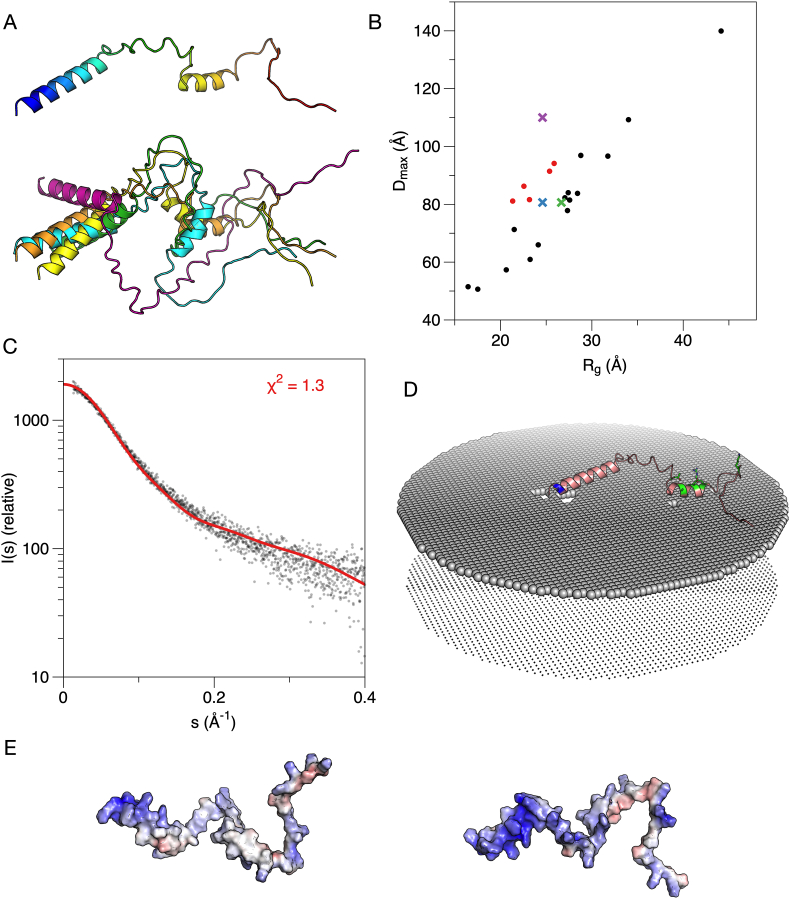
**AlphaFold2 analysis of P0ct.** A. The top-ranked model (top) and all five models superimposed (bottom). All models include two helices and have similar dimensions. B. Comparison of the P0ct AlphaFold2 models (red dots) and the full EOM ensemble (black dots). The green cross indicates the average values from EOM analysis of experimental data [50]. The Guinier R_g_ with D_max_ from EOM is marked with a blue cross and the Guinier R_g_ with manually determined D_max_ from GNOM with a magenta cross. C. The top ranked P0ct model fits the raw SAXS data very well. D. Docking of the P0ct model onto a membrane surface. Blue indicates the location of Cys182 close to the transmembrane domain, and CMT mutation sites are coloured green. E. Electrostatic surface of P0ct from two orientations. The face binding the membrane is hydrophobic (left), while the opposite side is positively charged (right). The entire P0ct is therefore predicted as amphipathic. (For interpretation of the references to colour in this figure legend, the reader is referred to the Web version of this article.)

P0ct AlphaFold2 models are shown in Fig. 5A, and their R_g_ and D_max_ distribution with respect to EOM results are shown in Fig. 5B. The outcome is similar to MBP, giving a further indication of the shared physicochemical properties (high positive charge, intrinsic disorder, folding upon membrane binding) between MBP and P0ct; for both, the AlphaFold2 model indicates more compact structure than experimentally measured in solution. One helix is predicted at the beginning of the P0ct; this segment is expected to bind along the membrane surface, and to be anchored to the membrane tightly *via* both the transmembrane domain and the palmitoylated Cys182 [31]. Earlier SRCD work has shown the corresponding synthetic peptide, with a thiopalmitoyl modification on Cys182, to fold into helical conformation in the presence of lipid membranes [25,31]. A second helix is in the middle region of P0ct and represents an additional membrane anchor [49]; whether it binds to the same or the apposing membrane in myelin, is currently not known. Mutations D224Y and R227S at this helical site are linked to CMT [[43], [44], [45], [46],^49^,^50^]. Intriguingly, this site is also a hotspot for PTMs, such as phosphorylation, that have been linked to the trafficking of P0 during myelination [42].

The highest-ranked AlphaFold2 model of P0ct fits reasonably well to the SAXS data (Fig. 5C). OLIGOMER fitting of the 5 top models did not improve this fit, and the full EOM ensemble fit slightly better than a single predicted model, indicating that the model approximates the average size and shape of P0ct in solution. Furthermore, the original *ab initio* model built based on the SAXS data [50] again provides a slightly better fit than the AlphaFold2 model (Table 1).

The helices predicted by AlphaFold2 on P0ct coincide with earlier identified functional segments interacting with lipid membranes. Furthermore, peptides encompassing the predicted helices in P0ct have been used to generate animal models of human autoimmune neuropathies [81]. Hence, these sites are known to show disorder-to-order transitions and either carry point mutations for CMT disease or autoantigenic epitopes that induce experimental autoimmune neuritis and possibly human Guillain-Barré syndrome [49,82].

Interestingly for an IDP, a total of 6 missense mutations linked to human CMT have been identified in the P0ct [[43], [44], [45], [46], [47], [48]]. The location of these mutations in the model is depicted in Fig. 5D, which also shows the predicted orientation of P0ct on a membrane surface. Importantly, these mutations are concentrated within the central region of P0ct, mainly in the membrane-binding helix. One of them, D224Y, causes both hypermyelination in patients and increased membrane stacking *in vitro* [45,49]. The model suggests the CMT mutations in P0ct could directly affect its membrane interactions in the tightly confined space of the myelin major dense line. The electrostatic potential surface of the P0ct highest ranked model is shown in Fig. 5E, indicating a positively charged and a hydrophobic face, compatible with amphipathic membrane interactions. Considering the tightly confined space of the PNS major dense line, we currently do not know whether the middle helical segment of P0ct binds to the same membrane as the transmembrane domain and the first helix of P0ct, or if it reaches over and inserts itself into the apposing cytoplasmic leaflet.

### 3.3 SRCD data indicate the AlphaFold2 models correspond to the membrane-bound form

**Fig. 6:**
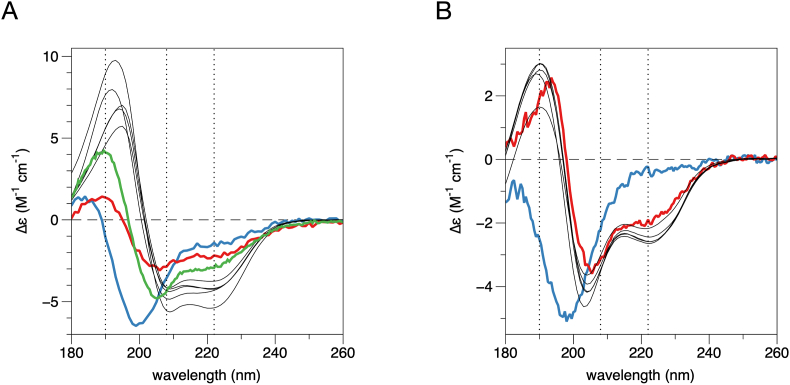
**Experimental SRCD spectra vs. predictions.** A. SRCD spectra for P0ct measured in water (blue), SDS micelles (green), and DMPC:DMPG liposomes (red). Note that the spectrum with liposomes is weaker than expected due to light scattering from stacked membrane particles. The calculated spectra from the AlphaFold2 models using PDB2CD are shown as black thin lines. B. SRCD spectra for MBP measured in water (blue) and DMPC:DMPG (red). PDB2CD spectra as in (A). (For interpretation of the references to colour in this figure legend, the reader is referred to the Web version of this article.)

As discussed above, comparison of the 3D AlphaFold2 models to SAXS data in solution suggested that the models could represent the membrane-bound conformation, rather than the conformational ensemble in solution. Hence, we also compared the models to SRCD data with and without lipid membranes. To analyse the folding of MBP and P0ct, the AlphaFold2 models were given as input to the PDBCD prediction tool [60]. The predicted CD spectra were compared to published experimental SRCD data [83,50] (Fig. 6).

For both proteins, PDB2CD produces spectra matching helical structures; a positive peak at 190 nm and negative minima at 208 and 222 nm. Experimental data of either protein in solution have the characteristic shape of an IDP, while the predicted spectra all show a partially helical conformation. The DMPC:DMPG (1:1) spectrum of P0ct is weak, probably due to light scattering in the presence of large particles, but it has the same minima and maxima as the spectrum measured in the detergent SDS (Fig. 6A), indicating helical structure. The five models of MBP fit the experimental SRCD spectra of MBP in DMPC:DMPG (1:1) liposomes quite well (Fig. 6B). Taken together, comparison of the AlphaFold2 models of MBP and P0ct to experimental SRCD data indicates that the folding state resembles that caused by lipid membrane binding.

### 3.4 Confidence scores for the models

AlphaFold2 gives as output per-residue confidence score (pLDDT) and predicted aligned error (PAE), crucial for interpreting the models. Because all predicted models of P0ct and MBP showed helices at similar positions in the sequence, one model from each protein was subjected for further analyses (Supplementary Figures 1,2).

Alderson et al. [15] discuss that AlphaFold2 assigns high pLDDT scores to IDRs that conditionally fold, and that the AlphaFold2 model may represent one folded conformation of the IDP(R) in the presence of a specific binding partner or upon PTMs. They hypothesized that IDR with pLDTT <50 reflect amino acid compositions found in disordered regions, and for IDR with pLDDT ≥70, ordering-promoting residues would dominate [84]. This holds for both MBP and P0ct. The predicted helical segments contain an increased number of amino acids promoting folding. The types of amino acids in the different regions modelled by AlphaFold2 agree with the amino acid biases found in disordered regions [15,84].

For P0ct (Supplementary Fig. 1A), AlphaFold2 predicts the first helix at Tyr181-Lys199, with a pLDTT >90, *i.e.* very high confidence. For helix 2 (Val218-Ser228), pLDDT is somewhat lower. A helix with low pLDDT score might be more transient than a helix with higher pLDDT, possibly providing insight into underlying local dynamics []. The PAE indicates that both P0ct helices are reliable in a local context, but their position with respect to each other is uncertain. At the C-terminal end, pLDDT <50 is a strong predictor of disorder, suggesting that this region is unstructured under physiological conditions.

For MBP (Supplementary Fig. 2A), pLDDT >70 for the three helices, with helix 2 having the highest scores; this segment is the best-characterised membrane-binding segment in MBP. When looking at the PAE and pLDDT scores together, the arrangement of the first and second helix with respect to one another is considered reliable, while there is no correlation with the position of the last helix. For the disordered regions, the pLDDT scores are low (<70) and the PAE score is high, indicating uncertain structure prediction. Looking at the sequence bias [84] for both MBP and P0ct, all predicted helices have more amino acids that are order-promoting, while the predicted disordered segments contain more disorder-promoting residues (Supplementary Fig. 1B, 2B).

AlphaFold2 predicts one specific structure, but IDPs have a high degree of conformational flexibility, which allows them to interact with multiple binding partners [18,85,86]. It is probable that both MBP and P0ct form “fuzzy” complexes with membrane surfaces, *i.e.* the disordered regions outside the membrane-attached helices would remain dynamic and flexible. Different ways of forming fuzzy complexes can be envisioned, and one possibility is a clamp-like interaction of two helices with a membrane surface, while not necessarily coming into contact with each other [67]. The two first helices of MBP are predicted to lie close to each other in 3D space, as shown by the PAE plots. This could involve cooperative binding of the helices, as theoretically described [87]. Whether this can happen on the membrane surface, and whether the MBP helices truly interact with each other in the lipidic environment, remains a subject for further research. As the membrane surface carries a high local MBP concentration, such cooperative interactions could also be intermolecular.

### 3.5 Hydrophobic cluster analysis provides further evidence for helical segments

AlphaFold2 models have been discussed in the light of hydrophobic cluster analysis (HCA). Bruley et al. investigated HCA from 21 proteomes, looked at the 3D predictions from AlphaFold2, and considered the low pLDDT scores given for disordered regions, revealing that predicted disordered regions that contain hydrophobic clusters (HC) most likely contain either conditional order or hidden, non-conditional order [23]. In the case of MBP and P0ct, such HCs are in fact predicted to be helical, indicating a high propensity to fold upon lipid membrane binding.

In HCA, a protein sequence is translated into a binary sequence, using a HC-specific code (1 = VILFMYW, * = P, 0 = other residues). HCs always begin and end with “1”. The Peitsch code (P-code) is defined as the decimal conversion of the binary code [88]. A high P-code represents a HC strongly associated with secondary structures. High density in HCs indicates the presence of foldable regions [89,90]. Residues in proximity of HCs also affect secondary structure formation. Ala and Leu have a preference for α-helices, while Gly and Cys correlate more with a β-strand [89].

**Fig. 7:**
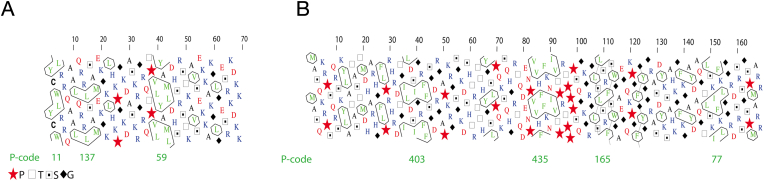
**Hydrophobic cluster analysis.** A. HCA for P0ct; note that residue numbering starts at the beginning of P0ct, corresponding to P0 residue 180. B. HCA for MBP. The Peitsch codes (P-codes) below the HCA indicate the main HCs in both proteins. HC maps were drawn using DrawHCA (http://osbornite.impmc.upmc.fr/hca/hca-form.html) [91].

The HCs found in P0ct correspond to the helices predicted by AlphaFold2 (Fig. 7A). The predicted α-helices are longer than the HCs; the helical segments have Ala residues, which contribute to α-helix extension outside the HC limits [91]. For the predicted disordered regions, many residues known to promote disorder are found, for instance Lys and Glu. In P0ct, two Pro residues are found between the predicted helices. Pro cannot stabilize α-helices or β-sheets, and when found within helical segments, it causes kinks [92], which are important in proteins that needs to adapt to their molecular environment, such as membrane proteins [93]. In P0ct, Pro217 right before the second helix could help orient the following secondary structure upon binding to membranes. A tilted conformation of this helix was detected on a lipid membrane using oriented CD spectroscopy [50].

AlphaFold2 predicted MBP to have three helical segments and the P-code of the first two (Ile36-Ser45 and Pro83-Val92) are highly indicative of secondary structures (Fig. 7B). Both have also strong propensity towards helical conformation according to the APC/OPS table [89]. For the last helix (Thr148-Phe153), the HC is shifted towards the end of the predicted helix. Each of the disordered regions contain at least one Pro. For instance, the segment 82–93 of MBP has prolines both before and after, and this helix is tilted when bound to a membrane [36]; this could be promoted by the Pro residues.

### 3.6 Additional notes on fitting to SAXS data

For both MBP and P0ct, EOM gives the best fit to the experimental SAXS data. Single *ab initio* models fit the data slightly worse than the full conformational EOM ensembles, suggesting the presence of several conformations. In the case of P0ct, a single AlphaFold2 model fits to the solution SAXS data from recombinant P0ct, which might suggest that the model is close to the average of the conformational ensemble. However, as can be seen from the plot of D_max_ vs. R_g_, this is not the case (Fig. 5B). IDPs are often not straightforward cases for SAXS studies, as discussed in recent literature [94]. The data do indicate that single models of IDPs from AlphaFold2 can complement SAXS data and provide an estimate of the size and shape of the IDP at low resolution, possibly in the bound state. As for folded proteins, therefore, such models can be valuable additions to support experimental data and help in setting up and evaluating hypotheses on structure-function relationships.

In essence, for both IDPs studied here, the AlphaFold2 models are close to the average D_max_ of the disordered EOM ensemble and the R_g_ obtained from Guinier plot (Fig. 3, Fig. 5B). On the other hand, D_max_ determined in a traditional way subjectively from distance distribution more estimates the absolute largest D_max_ in the population instead of the average (Fig. 3, Fig. 5B). For IDPs, Debye formalism provides a more relevant R_g_ than the Guinier plot [57], and indeed, this value is close to that of the EOM ensemble average R_g_. From Debye analysis, the R_g_ for MBP is 42.1 Å and that for P0ct 26.2 Å. These analyses further indicate that the AlphaFold2 models do not appear to represent the disordered ensembles in solution, but systematically show slightly more compacted conformations, possibly corresponding to the lipid-bound conformation. The latter is also strongly suggested by the corresponding SRCD spectra of the membrane-bound states of both proteins.

### 3.7 IDPs and IDRs related to human neuropathies

Changes that disrupt the structural integrity and function of the myelin sheath result in neurological diseases, such as multiple sclerosis, various types of CMT, Dejerine-Sottas syndrome, and congenital hypomyelination. These in turn may arise *e.g.* from autoimmune reactions against MBP, as well as mutations in myelin proteins. Intriguingly, many mutations linked to human peripheral neuropathies, mainly different forms of CMT, are found in IDPs or IDRs. This highlights the important functional/structural role of these protein segments, whereby they may be important membrane interaction sites or participate in protein-protein complexes. CMT mutations are found in both P0ct [[43], [44], [45], [46], [47], [48]] and in the extended disordered regions of periaxin [[95], [96], [97], [98]]. For P0ct, such mutations are expected to affect protein-membrane interactions [49] or to cause P0 retainment in the ER during expression [99]. In periaxin, the mutations disturb protein-protein interactions. In line with this, disease mutations affecting IDRs often cluster at sites of protein-protein interactions [100]. Simulations have additionally suggested that disease mutations may reduce conformational heterogeneity of IDRs, which may in turn affect protein interactions [101].

Puzzlingly, thus far, no mutations in MBP have been linked to any human disease, despite its high abundance and apparent importance for myelin structure; however, there is evidence to suggest that its main autoantigenic epitopes correspond to its membrane binding sites [25,62,102]. Also for other myelin proteins, membrane binding segments that fold into helices [25,26,31] are highly antigenic and can be used to induce autoimmune disease in animal models. For MBP, there is evidence suggesting that PTMs, while allowing for MBP binding to membranes, affect the overall conformation and dynamics of the membrane-bound state [103]. Aberrant citrullination of MBP has been linked to dysmyelination and multiple sclerosis [[104], [105], [106]]. Furthermore, the membrane-binding segments of MBP have been predicted to be sites of conditional folding, using sequence-based bioinformatics [62]. Another application for AlphaFold2 could, thus, be to provide information on putative autoantigenic epitopes on autoimmune disease-linked IDPs.

## 4 Conclusions

The intermembrane compartment harbouring MBP and P0ct in the PNS, the major dense line, is very tight, with a spacing of only ∼3 nm between the bilayers. This indicates, together with the expected molecular dimensions of both MBP and P0ct, that both proteins must interact with two membranes simultaneously and go through some form of compaction. These interactions are enabled by both the membrane anchor segments forming α-helices as well as the flexible, disordered segments between them. The use of AlphaFold2 models in this short report has highlighted that molecular models can be used to obtain additional details of functional significance in combination with experimental data. In some cases, conclusions can be drawn, for example, on the effects of disease mutations on IDP structure and interactions. While the overall 3D structure of a single AlphaFold2 model of an IDP will not be accurate, nor does it give much information about conformational ensembles, it does give relevant information about average molecular size and shape, as well as segments that are likely to fold into secondary structure upon molecular interactions. Accordingly, it has not escaped our attention that for both of the myelin IDPs studied here, the AlphaFold2 models, while at first glance fitting to the solution SAXS data reasonably, considering the only input to modelling was the sequence of an IDP, compare remarkably well with the SRCD data of the membrane-bound forms. Hence, AlphaFold2 does provide meaningful information on at least the overall size and shape of these IDPs, but it additionally has the power to predict interaction sites and conditionally folded segments linked to them. In combination with experimental biophysical and structural work on IDPs, the predicted models can help explain molecular mechanisms in IDP biology and disease.

## Funding

This work was funded through a FRIPRO grant (324877) from the Research Council of Norway (to PK).

## Author contributions

OCK: conceptualization, investigation, writing – original draft. AR: data curation, investigation, methodology, resources, supervision, validation, writing – review & editing. PK: conceptualization, formal analysis, funding acquisition, project administration, supervision, visualization, writing – original draft.

## Declaration of competing interest

The authors declare that they have no known competing financial interests or personal relationships that could have appeared to influence the work reported in this paper.

## Data Availability

Data will be made available on request.

## Appendix A Supplementary data

Supplementary Fig. 1 **Confidence scores and amino acid composition for P0ct AlphaFold2 models**. A. Top: Structure of the highest-ranked model coloured by pLDDT. Bottom left: pLDDT plot. Bottom right: PAE plot. B. Number of amino acids in the different Alphafold2 regions found to promote either order or disorder. Disordered region 1 is only one residue before the first helix. Supplementary Fig. 1

Supplementary Fig. 2 **Confidence scores and amino acid composition for MBP AlphaFold2 models**. A. Top: Structure of the highest-ranked MBP model coloured by pLDDT. Bottom left: pLDDT plot. Bottom right: PAE plot. Note the cross-correlation between the first and second helices in the MBP model. B. Number of amino acids in the different AlphaFold2 regions found to promote either order or disorder. Supplementary Fig. 2
