# Supplementary figures and images for: The intrinsically disordered protein glue of the myelin major dense line: Linking AlphaFold2 predictions to experimental data
Source: Biochem Biophys Rep. 2023 Apr 26;34:101474. doi: 10.1016/j.bbrep.2023.101474 (PMC10160357; doi:10.1016/j.bbrep.2023.101474)

A

P0ct

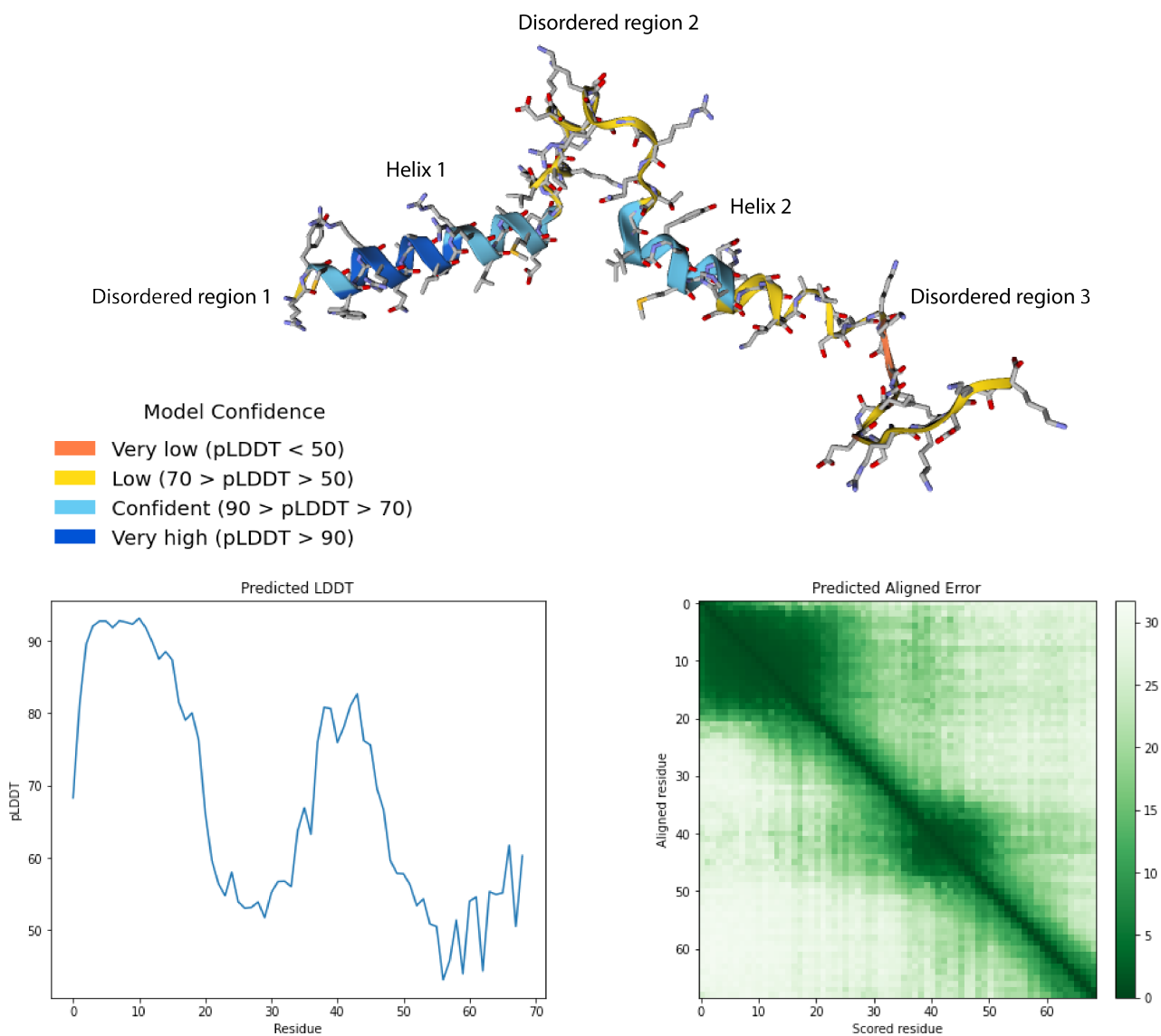

B

P0ct

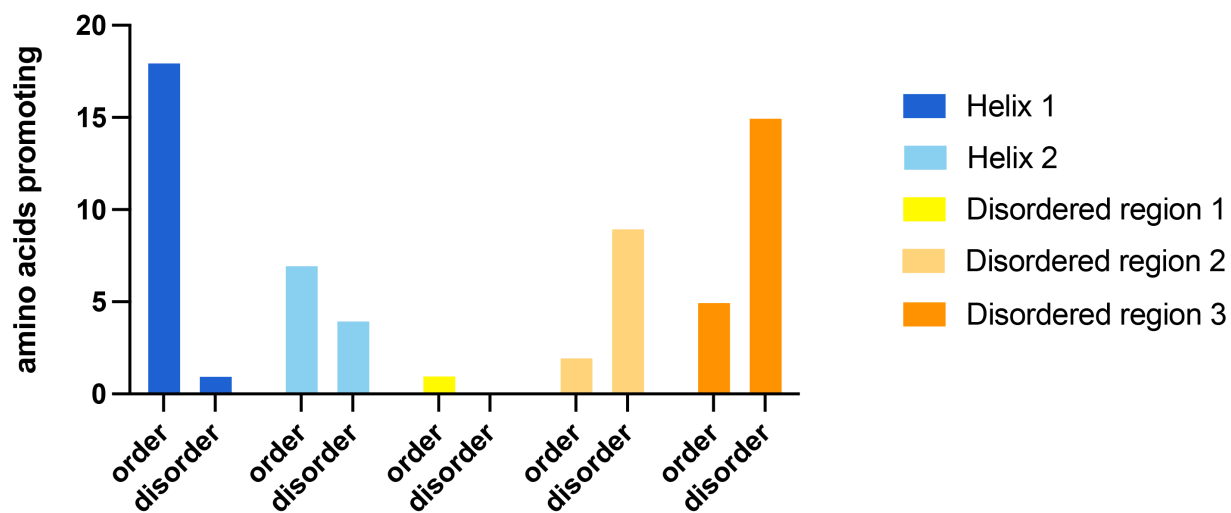

Supplement: Supplementary Fig. 1 — Confidence scores and amino acid composition for P0ct AlphaFold2 models. A. Top: Structure of the highest-ranked model coloured by pLDDT. Bottom left: pLDDT plot. Bottom right: PAE plot. B. Number of amino acids in the different Alphafold2 regions found to promote either order or disorder. Disordered region 1 is only one residue before the first helix. [file mmc1.pdf]

A

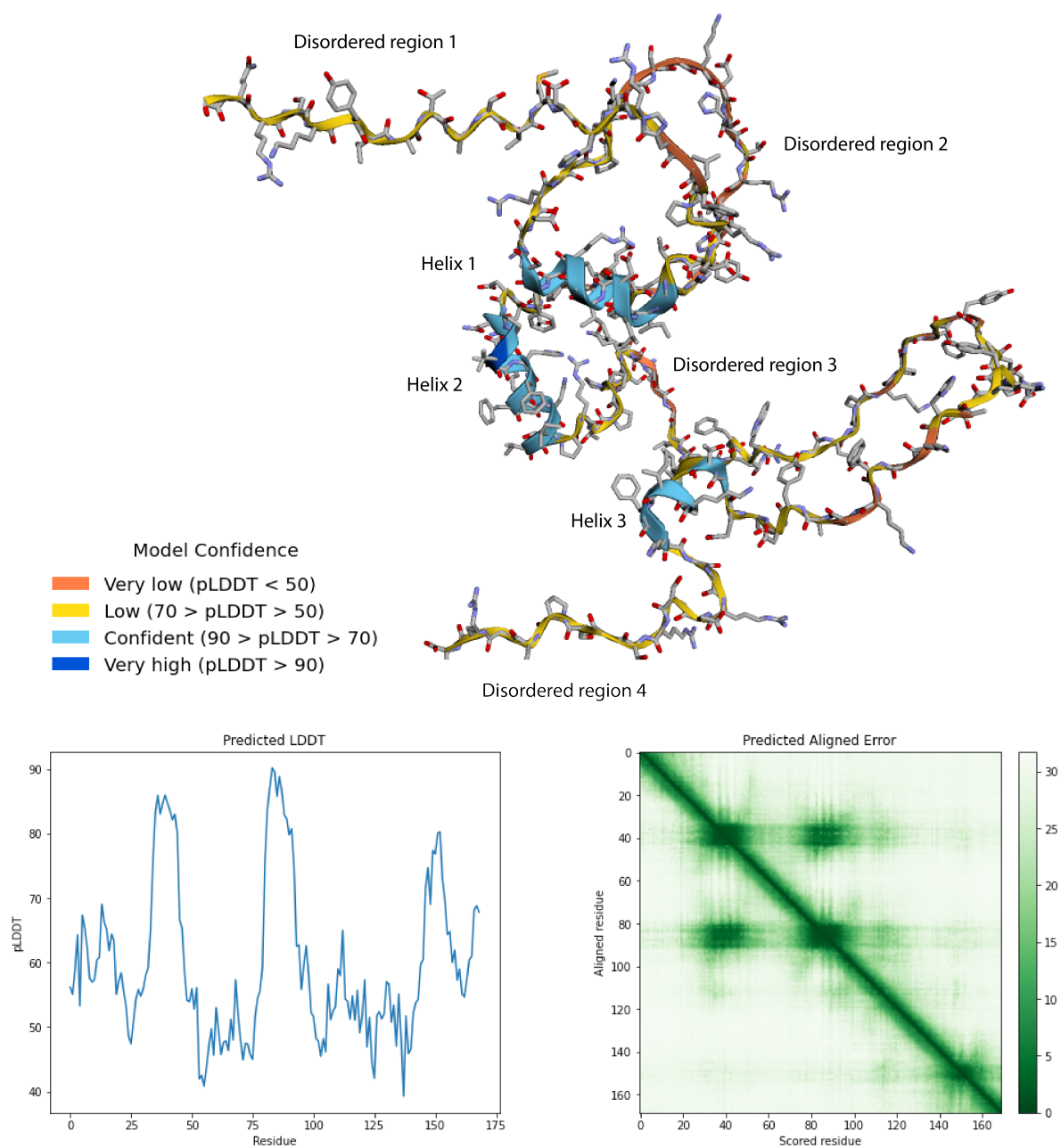

B

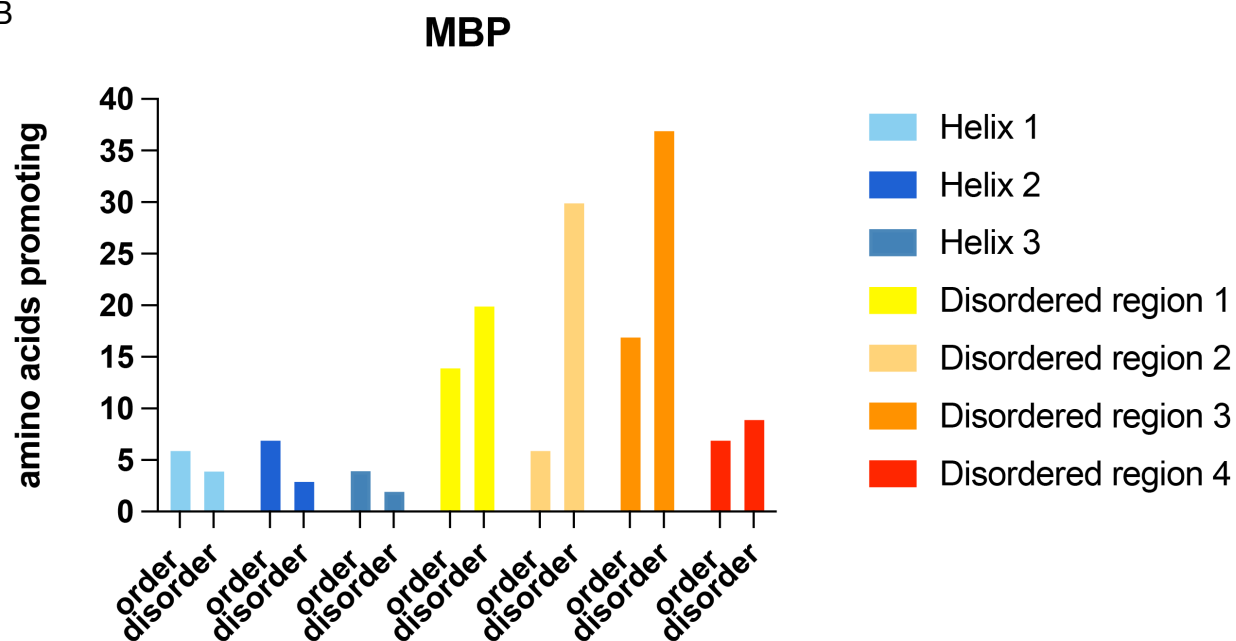

Supplement: Supplementary Fig. 2 — Confidence scores and amino acid composition for MBP AlphaFold2 models. A. Top: Structure of the highest-ranked MBP model coloured by pLDDT. Bottom left: pLDDT plot. Bottom right: PAE plot. Note the cross-correlation between the first and second helices in the MBP model. B. Number of amino acids in the different AlphaFold2 regions found to promote either order or disorder. [file mmc2.pdf]
